# Distinct noncoding RNAs and RNA binding proteins associated with high‐risk pediatric and adult acute myeloid leukemias detected by regulatory network analysis

**DOI:** 10.1002/cnr2.1592

**Published:** 2021-12-04

**Authors:** Zhenqiu Liu, Vladimir S. Spiegelman, Hong‐Gang Wang

**Affiliations:** ^1^ Department of Public Health Sciences Pennsylvania State University College of Medicine Hershey Pennsylvania USA; ^2^ Division of Pediatric Hematology and Oncology, Department of Pediatrics Penn State College of Medicine Hershey Pennsylvania USA

**Keywords:** adult and pediatric AMLs, distance correlation test, gene regulatory networks, high‐risk AMLs, ncRNAs, prognostic markers, RBPs

## Abstract

**Background:**

Acute myeloid leukemia (AML) is a heterogeneous disease in both children and adults. Although it is well‐known that adult and pediatric AMLs are genetically distinct diseases, the driver genes for high‐risk pediatric and adult AMLs are still not fully understood. Particularly, the interactions between RNA binding proteins (RBPs) and noncoding RNAs (ncRNAs) for high‐risk AMLs have not been explored.

**Aim:**

To identify RBPs and noncoding RNAs (ncRNAs) that are the master regulators of high‐risk AML.

**Methods:**

In this manuscript, we identify over 400 upregulated genes in high‐risk adult and pediatric AMLs respectively with the expression profiles of TCGA and TARGET cohorts. There are less than 5% genes commonly upregulated in both cohorts, highlighting the genetic differences in adult and childhood AMLs. A novel distance correlation test is proposed for gene regulatory network construction. We build RBP‐based regulatory networks with upregulated genes in high‐risk adult and pediatric AMLs, separately.

**Results:**

We discover that three RBPs, three snoRNAs, and two circRNAs function together and regulate over 100 upregulated RNA targets in adult AML, whereas two RBPs are associated with 17 long noncoding RNAs (lncRNAs), and all together regulate over 90 upregulated RNA targets in pediatric AML. Of which, two RBPs, MLLT3 and RBPMS, and their circRNA targets, PTK2 and NRIP1, are associated with the overall survival (OS) in adult AML (*p* ≤ 0.01), whereas two different RBPs, MSI2 and DNMT3B, and 13 (out of 17) associated lncRNAs are prognostically significant in pediatric AML.

**Conclusions:**

Both RBPs and ncRNAs are known to be the major regulators of transcriptional processes. The RBP–ncRNA pairs identified from the regulatory networks will allow better understanding of molecular mechanisms underlying high‐risk adult and pediatric AMLs, and assist in the development of novel RBPs and ncRNAs based therapeutic strategies.

## 1 INTRODUCTION

Acute myeloid leukemia (AML) is a heterogeneous disease in both children and adults. It is the most common acute leukemia caused by malignant transformation of hematopoietic progenitor cells through a wide range of molecular alterations.^1^ The risk of developing AML is also age‐associated with the number of incidences rising with age, and childhood AML is different from adult AML both biologically and clinically.^2^, ^3^ In general, pediatric AML has a lower number of somatic mutations and higher frequency of cytogenetic abnormalities compared to its adult counterpart.^4^, ^5^, ^6^ Besides their significant differences in genetic landscape, there are also vast differences in their epigenetic landscape, leading to distinct expression patterns in childhood and adult AML.^7^ In addition, the prognoses of childhood and adult AML are quite different. The overall survival (OS) in adults remains low with the 5‐year survival rate of 20%. While the OS in children is higher with the 5‐year survival rates of 70%, However, intensive chemotherapy regimens in children are burdensome, and half of childhood leukemia‐related deaths are caused by relapsed/refractory AML.^7^, ^8^ Particularly, different pediatric AML subtypes (low, intermediate, and high‐risk) have very different prognoses with the 5‐year survival ranging from 22 to 90%, and the 5‐year OS for high‐risk pediatric AML is below 30%.^9^, ^10^ Few new drugs for pediatric AML have been discovered over the decade, despite biological and technical advances. There is an urgent need for better therapies for both pediatric and adult AML.

RNA binding proteins (RBPs) are proteins that bind RNA with or without RNA‐binding domains (RBDs) and play a pivotal role in post‐transcriptional regulation of gene expression.^11^, ^12^, ^13^ RBPs regulate various aspects of RNA function, including transcription, splicing, modification, intracellular trafficking, translation and decay.^13^, ^14^ A RBP can bind and control a large number of RNA targets, and is involved in dynamic interactions of RBPs and their regulated RNAs .^15^ The deregulations and malfunctions of RBPs may lead to many diseases including cancers.^16^ In AML, aberrant RBP expression has been commonly linked to promoting cancer progression through co‐ and post‐transcriptional mechanisms.^17^ However, although it is promising to target RBPs therapeutically for AML,^18^, ^19^ the progression in drug development with RBPs has been limited.

Next generation sequencing technologies with genomes and transcriptomes have demonstrated that less than 2% of the entire human genome encodes proteins, but up to 80% of it can be transcribed into noncoding RNAs (ncRNAs). ncRNAs are the transcripts that do not code for functional proteins.^20^ ncRNAs are classified into two broad categories according to transcript size. Noncoding RNAs with less than 200 nucleotides (nt) in length are considered as small ncRNAs (sncRNAs), whereas ncRNAs with more than 200 nt are long ncRNAs (lncRNAs).^21^, ^22^ Small ncRNAs can be further divided into several different types of ncRNAs including microRNAs (miRNAs), small nucleolar RNAs (snoRNAs), and others.^23^, ^24^ lncRNAs can originate from different genomic locations, and also exhibit diverse structures with different subclasses including linear lncRNAs and circular RNAs (circRNAs).^25^, ^26^ Circular RNAs (circRNAs) are closed long non‐coding RNAs, in which the 5′ and 3′ termini are covalently linked by back‐splicing of exons from a single pre‐mRNA, and have been shown to be involved in regulating important biological processes .^26^, ^27^ The ncRNAs as a whole play pivotal roles in the process of gene expression, RNA maturation, and protein synthesis, and may control gene expression and disease progression. However, although there are separate studies that ncRNAs, or RBPs are involved in the pathogenesis of AML through regulating gene expressions,^17^, ^28^, ^29^, ^30^ the crosstalk between RBPs and ncRNAs in AML has not been studied. The regulatory interactions between RBPs and ncRNAs are insufficiently understood in both childhood and adult AMLs.

To dissect the roles of ncRNAs and RBPs in adult and pediatric AML, and explore the potentials of targeting RBPs or ncRNAs therapeutically, we must study the complex regulatory networks formed by the interactions among RBPs, ncRNAs, and targeted coding RNAs, and fully understand the molecular mechanisms of the RBP and ncRNA functions in AML. Therefore, in this pilot project, we are concentrating on building gene regulatory networks with adult and childhood AML transcriptomic data, and identify RBPs, ncRNA, and their interactions that are important for high‐risk adult and pediatric AMLs, respectively.

## 2 MATERIALS AND METHODS

### 2.1 Datasets

#### 2.1.1 TCGA adult AML data

The RNA‐seq transcriptomic profile and corresponding clinical metadata from the TCGA project are downloaded from cbioportal (https://www.cbioportal.org/).^31^ There are a total of 173 samples with 20 531 raw gene counts available. Patients were originally divided into three risk subgroups including good (low), intermediate (standard), and poor (high) risk subtypes based on the cytogenetic classification. The ages of the patients range from 18 to 88 years old. Both overall and progress free survival data are available. The raw counts are normalized with log2 transformation and quantile normalization.

#### 2.1.2 TARGET pediatric AML data

RNA‐seq data for pediatric AML are downloaded from the Genomic Data Commons (GDC; portal.gdc.cancer.gov/).^32^ The TARGET pediatric AML cohort consists of 187 samples with tissues including primary bone marrow (*N* = 119), recurrent bone marrow samples (*N* = 40), primary peripheral blood (*N* = 26), and recurrent peripheral blood (*N* = 2). Both raw gene counts and clinical metadata are available. The raw transcriptomic counts were originally produced using the Illumina Hi‐Seq platform from the Genomic Data Commons repository (https://gdc.cancer.gov/). The raw reads were aligned to GRCh38 using STAR aligned in 2‐pass mode and gene counts were produced using the HTSeq‐counts analysis workflow with Gencode v22 annotations. The data processing pipeline can be found at the GDC website (https://docs.gdc.cancer.gov/Data/). The patients in this cohort also have 3 risk groups (Low, Intermediate, and High). There are 21 047 genes with nonzero reads. The raw data was normalized with a trimmed mean of M (TMM) values method and converted to log2 counts per million by Reference 33.

### 2.2 Distance correlation test for RBP‐based gene regulatory network analysis

RBPs bind and control a wide array of RNA targets that are critical for cancer progression. Most recently, there has been evidence that RBPs act as important regulators of lncRNAs in cancer.^34^ Mechanically, RBPs are commonly deregulated in cancer and might thus play a major role in the deregulation of cancer‐related ncRNAs. However, it is not clear how RBPs interact with ncRNAs in pediatric and adult AML. We, therefore, aim to construct RBP‐based gene regulatory networks with distance correlation tests, and explore RBP and ncRNA interactions in high‐risk AMLs.

Distance correlation was proposed recently to measure both linear and nonlinear dependence between two sets of variables.^35^, ^36^ It is straightforward to compute and asymptotically equals zero if and only if independence. Given the expressions of the paired of genes (*x*,*y*) = {(*x*_*i*,_ *y*_*i*_)*, i* = 1,2,…,*n*}, where *x* denotes a RBP, and *y* represents its coding RNA or ncRNA targets, and *n* is the number of patient samples, the distance matrices *A* and *B* for *x* and *y*, respectively, are defined as $A={\left[a_{\mathrm{ij}}\right]}_{n\times n}$ where $a_{\mathrm{ij}}=\left|x_{i}-\left.x_{j}\right|\right.$, and $B={\left[b_{\mathrm{ij}}\right]}_{n\times n}$ where $b_{\mathrm{ij}}=\left|y_{i}-\left.y_{j}\right|\right.$ with the Euclidean metric. The unbiased distance matrix $A^{\left(u\right)}$ for *x* is then estimated with

$$a_{\mathrm{ij}}^{\left(u\right)}=a_{\mathrm{ij}}-\frac{1}{n-2}\sum_{s=1}^{n}a_{\mathrm{sj}}-\frac{1}{n-2}\sum_{t=1}^{n}a_{\mathrm{it}}+\frac{1}{\left(n-1\right)\left(n-2\right)}\sum_{a,t=1}^{n}a_{\mathrm{st},}\text{if}\;i\neq j,\;\text{and}\;a_{\mathrm{ij}}^{\left(u\right)}=0,\;\text{if}\;i=j$$

Similarly, the unbiased distance matrix for is estimated with

$$b_{\mathrm{ij}}^{\left(u\right)}=b_{\mathrm{ij}}-\frac{1}{n-2}\sum_{s=1}^{n}b_{\mathrm{sj}}-\frac{1}{n-2}\sum_{t=1}^{n}b_{\mathrm{it}}+\frac{1}{\left(n-1\right)\left(n-2\right)}\sum_{s,t=1}^{n}b_{\mathrm{st},}\text{if}\;i\neq j,{\;\text{and}\;b}_{\mathrm{ij}}^{\left(u\right)}=0,\;\text{if}\;i=j$$

The sample distance covariance and variances are then estimated as

$${\mathrm{Cov}}_{D}\left(x,y\right)=\frac{1}{n\left(n-3\right)}\text{trace}\left(A^{\left(u\right)}B^{\left(u\right)}\right).$$

$${\mathrm{Var}}_{D}\left(x\right)=\frac{1}{n\left(n=3\right)}\text{trace}\left({\left(A^{\left(u\right)}\right)}^{2}\right),\;\text{and}\;{\mathrm{Var}}_{D}\left(y\right)=\frac{1}{n\left(n=3\right)}\text{trace}{\left(\left(B^{\left(u\right)}\right)\right)}^{2}$$

So the sample distance correlation can be calculated as

$$R_{D}\left(x,y\right)=\frac{{\mathrm{Cov}}_{D}\left(x,y\right)}{\sqrt{{\mathrm{Var}}_{D}\left(x\right){\mathrm{Var}}_{D}\left(y\right)}}\in \left[-1,1\right]$$

The associated RBP and its RNA target pairs are then identified with the Chi‐square statistical test,^36^ and the *p* value is calculated with

$$P=1-F_{x_{1}^{2}-1}\left({\mathrm{nR}}_{D}\left(x,y\right)\right)$$

We reject the null hypothesis that the RBP and its RNA target are independent if and only if is less than the significance level.

## 3 RESULTS

After filtering out the samples with missing risk group information, there are 37, 101, and 32 patients in the Poor (High), Intermediate (Standard), and Good (Low) risk groups in TCGA cohort, whereas there are 12, 93, and 72 subjects in the High, Standard, and Low risk groups in the TARGET cohort. We first compare the gene expression profile of high‐risk AML with the remaining risk groups (low + intermediate), and identify upregulated genes in adult and pediatric AMLs, respectively.

### 3.1 High‐risk pediatric and adult AMLs are genetically distinct diseases

**FIGURE 1:**
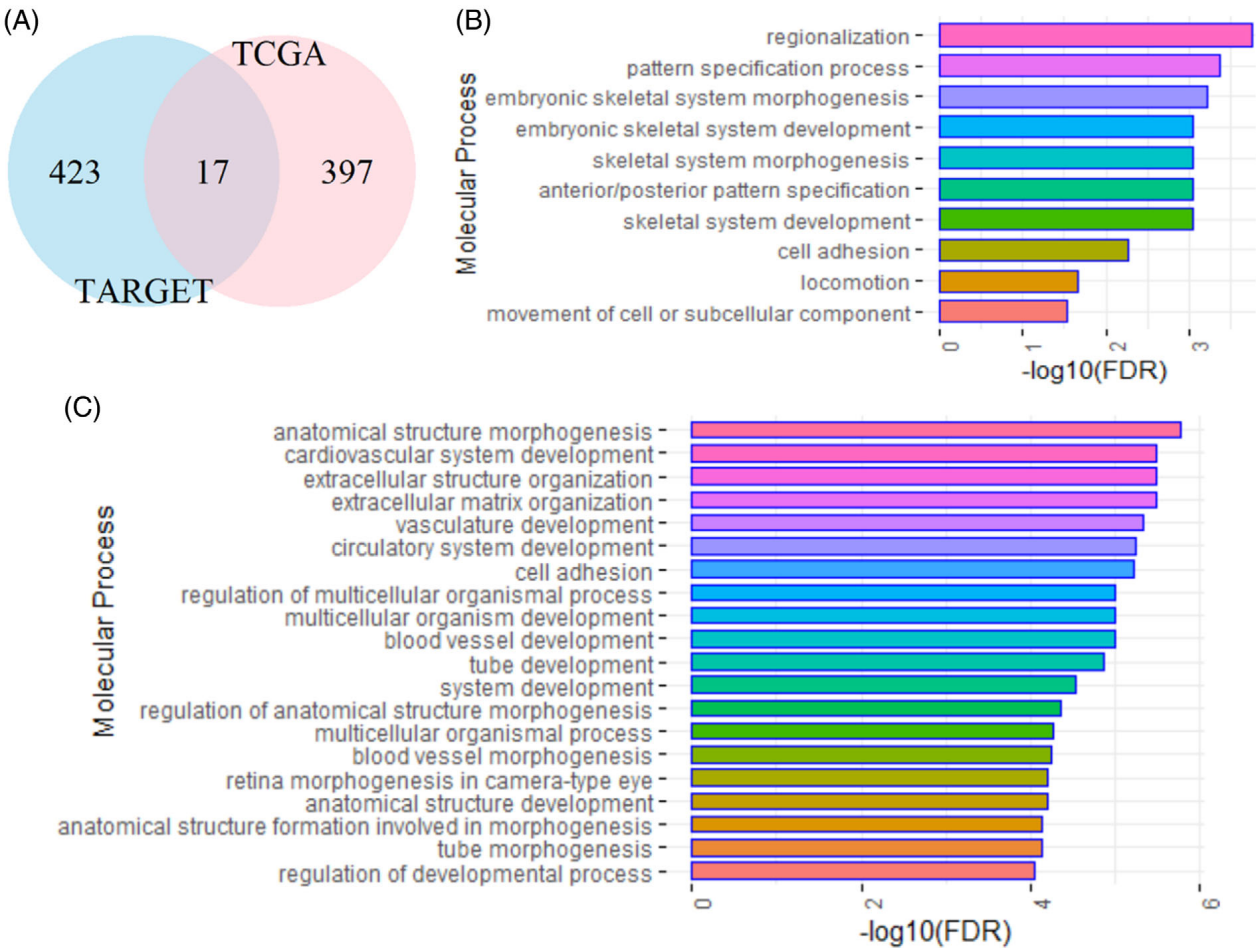
Venn diagram and gene enrichment analysis with STRING (https://string‐db.org/). (A) Venn diagram of the upregulated genes in high‐risk AML with the TARGET and TCGA cohorts. (B) Molecular process (gene ontology) with the upregulated genes in high‐risk pediatric AML with the TARGET cohort; (C) Molecular process (gene ontology) with the upregulated genes in high‐risk adult acute myeloid leukemia with the TCGA cohort

To prevent the confounding effect between the adult and childhood AML, we analyze their expression profiles separately. We perform a two‐sided Student's *T*‐test, and identify upregulated genes in high‐risk adult and pediatric AML, respectively, with the criteria of and two‐fold changes. A total of 414 upregulated genes in high‐risk adult AML are selected from the TCGA cohort, whereas 440 upregulated genes in high‐risk pediatric AML are discovered with the TARGET cohort. However there are only 17 (less than 5%) overlapped genes as demonstrated in Figure 1, and details of the identified genes are reported in Table S1.

Among the upregulated genes in high‐risk adult and pediatric AMLs, several of them have been investigated in previous publications. For instance, RGS10 and FAM26F were found to be prognostically significant in pediatric AML.^3^ Although RGS10 and FAM26F are not upregulated in TARGET cohort, we discover that one gene (RGS3) of the regulator of G‐protein signaling (RGS) family is upregulated, and several other members of Family With Sequence Similarity (FAM) including FAM30A, FAM124A, FAM47E, and FAM81A are also upregulated in childhood tumor. Moreover, several upregulated genes including CEACAM1, CEACAM6, CEACAM8 in TCGA, and HOXA3, HOXA5, HOXB5, and HOXB6 in TARGET were also found in,^37^ although the high‐risk subgroup was only compared to the low‐risk subtype in their study, Finally, previous study discovered promoter hypermethylation of genes CDH1 and WT1 in AML,^38^ we further demonstrate that CDH1 and WT1 are upregulated in adult AML.

As shown on the Venn diagram of the upregulated genes in Figure 1A, less than 5% of the upregulated genes from the TCGA and TARGET cohorts are overlapped, indicating that high‐risk pediatric and adult AMLs are genetically different. Among the 17 commonly upregulated genes in high‐risk adult and childhood AMLs, genes, CALCRL, CD59, DDIT4, and DOCK1, have been reported to be prognostically significant and potential markers.^39^, ^40^, ^41^, ^42^

The annotation and gene enrichment analysis of all upregulated genes was performed with the web resource of Search Tool for the Retrieval of Interacting Genes/Proteins (STRING:https://string-db.org/). The enriched molecular processes (gene ontology) of the upregulated genes in pediatric and adult AMLs are demonstrated in Figure 1B,C, respectively. The molecular pathways are significantly different. The top five molecular processes of the upregulated genes in high‐risk pediatric AML are involved in regionalization, pattern specification process, embryonic skeletal system morphogenesis, skeletal system development, and anterior/posterior pattern specification, whereas the top five molecular processes of the upregulated genes in high‐risk adult AML are anatomical structure morphogenesis, extracellular matrix organization, extracellular structure organization, cardiovascular system development, and vasculature development. Finally, more known molecular processes are involved in adult AML than in pediatric AML. Over 20 pathways are enriched in high‐risk adult AML, whereas only 10 known molecular processes are enriched in high‐risk childhood AML, suggesting the genetic differences in high‐risk adult and pediatric AMLs.

### 3.2 Upregulated RBPs in high‐risk adult and pediatric AMLs are also distinct

**FIGURE 2:**
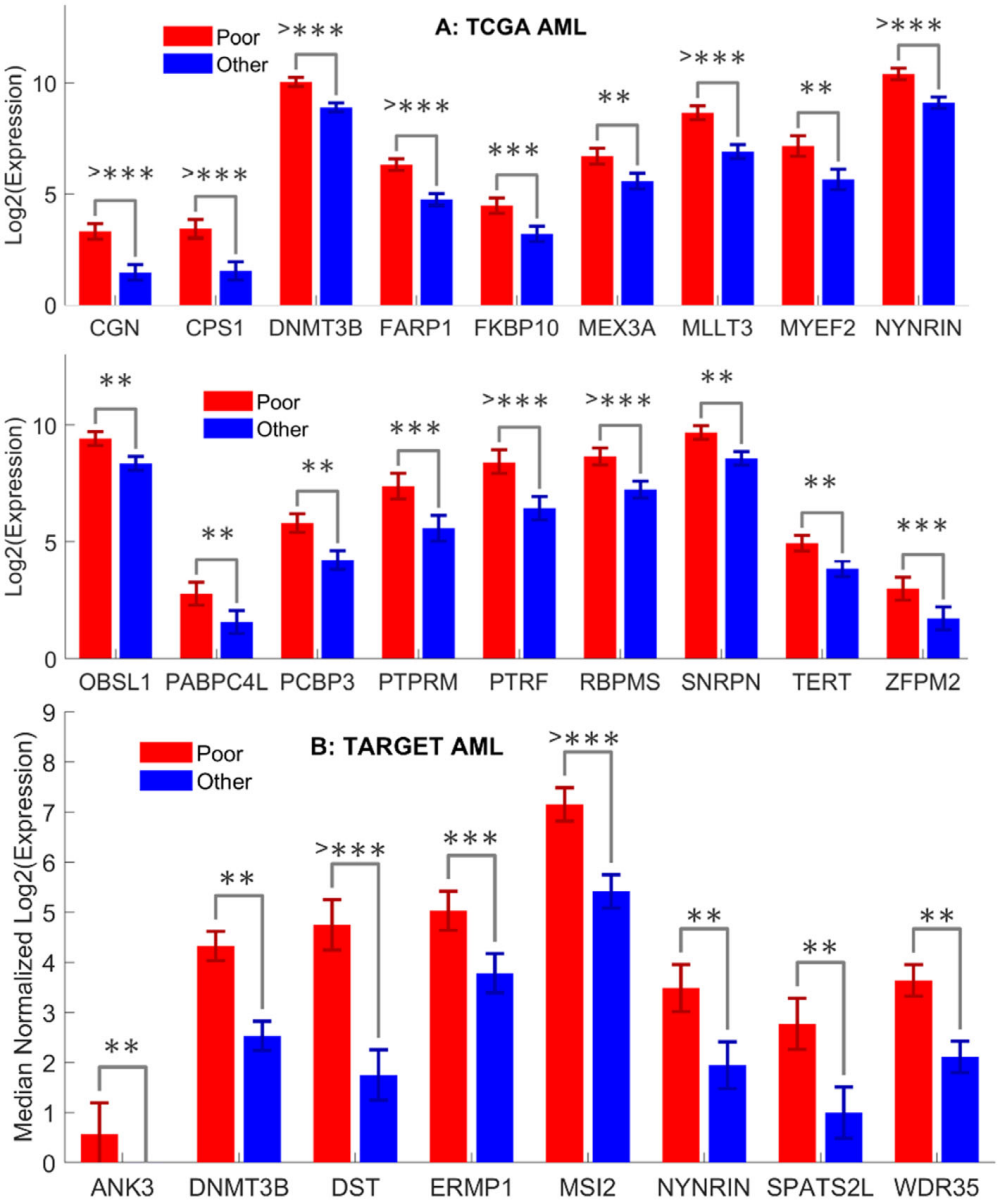
Upregulated RBPs in high‐risk adult and pediatric AML. (A) 18 upregulated RBPs in high‐risk adult AML. (B) 8 upregulated RBPs in pediatric AML, where the red bar denotes the expression in high‐risk AML and the blue bar represents the expression values in other (standard + low) risk groups. AML, acute myeloid leukemia; RBPs, RNA binding proteins

Out of 414 and 440 upregulated genes in high‐risk adult and pediatric AML, there are 18 and 8 upregulated RBPs in high‐risk adult and pediatric AML, respectively, as in Figure 2.

There are only two commonly upregulated RBPs including DNMT3B and NYNRIN out of 18 and 8 upregulated RBPs in high‐risk adult and pediatric AML, further indicating the differences in regulatory mechanisms. The two common RBPs, DNMT3B and NYNRIN, have been studied previously. DNMT3B (DNA Methyltransferase 3 Beta) is the enzymatic player of DNA methylation, and plays important roles in gene expression regulation and chromatin structure,^43^ while NYNRIN (NYN Domain And Retroviral Integrase Containing) is less studied. We discover that both genes are overexpressed in high‐risk AML, and the upregulations of two genes are associated with patient survival in both adult and pediatric AMLs.^44^ Next, we will construct RBP‐based gene regulatory networks with the upregulated genes in high‐risk adult and pediatric AML, respectively.

### 3.3 RBP and ncRNA pairs in adult AML identified through regulatory network analysis

**FIGURE 3:**
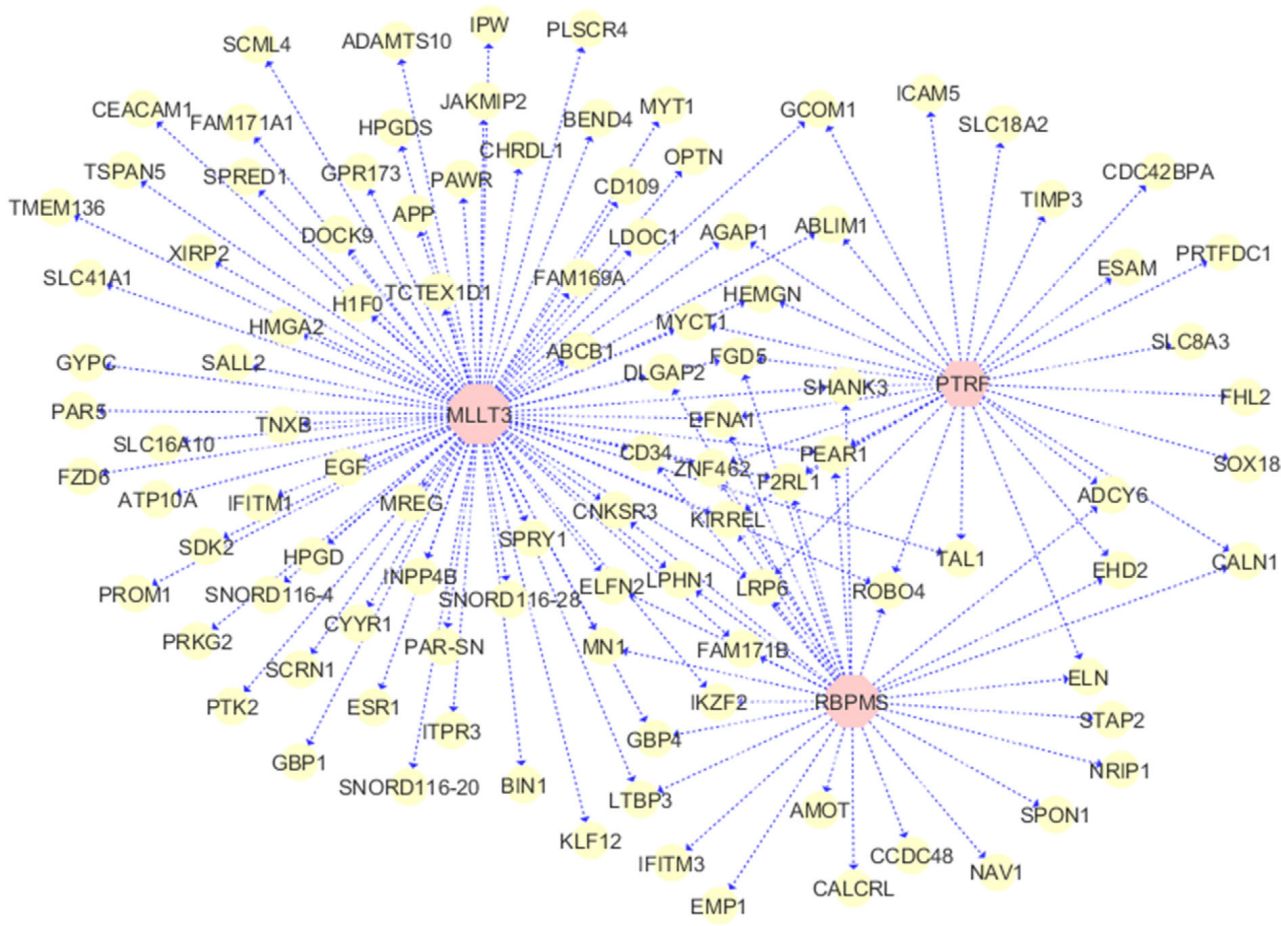
Gene regulatory network with upregulated RBPs and other RNAs in adult AML. The pink nodes at the center are the RBPs and the yellow nodes are the potential RNA targets. Only RBPs that interact with a large number of upregulated RNAs are included in the network. AML, acute myeloid leukemia; RBPs, RNA binding proteins

Distance correlation was used to construct gene regulatory networks with the 414 upregulated genes including 18 upregulated RBPs in the TCGA cohort. We concentrate only on the upregulated genes, because the upregulated genes are easier to be detected and validated in a wet laboratory, and the constructed network with those genes can have a reasonable size to be visualized. The interactions between RBPs and targeted RNAs are selected with raw $P<1e-06\;\left(R_{D}>0.2\right)$ by the Bonferroni correction. We identify 103 targeted RNAs regulated by three RBPs including MLLT3, RBPMS, and PTRF, as demonstrated in Figure 3.

MLLT3 is a RBP regulating the largest number of targeted genes (81 genes), while RBPMS and PTRF interact with 32 and 28 coding and noncoding RNAs, respectively. Note that there are nine RNA targets regulated by all three RBPs, and 29 genes are controlled by at least two RBPs, suggesting that multiple RBPs may co‐bind some of their targets. While it is well‐known that RBPs are essential modulators of transcription and translation, and the regulatory interplays of RBPs are required for AML survival,^17^ RBP functions are also known to be cell‐ and tissue‐specific. The same RBPs may bind to distinct RNA transcripts in different cell types, tissues, and tumor subtypes. We identify three novel RBPs and their targets specifically for high‐risk adult AML, More interestingly, the RBP MLLT3 interacts with three small nucleolar RNAs (snoRNAs) including SNORD116‐4, SNORD116‐20, and SNORD116‐28, which belong to the noncoding SNORD116 gene cluster. snoRNAs are mostly responsible for the posttranscriptional modification and maturation of ribosomal RNAs (rRNAs), gene transcription, RNA splicing, and other cellular processes.SNORD116 is considered to be orphan C/D box snoRNAs as it does not target rRNAs or snRNAs, but it is known to regulate the transcription of over 200 genes.^45^ snoRNAs are tissue‐ and cancer‐specific, and their roles in AML are still poorly understood. We discover that SNORD116‐4, −20, and −28 are upregulated in high‐risk adult AML and interact with MLLT3 to regulate a large number of genes post‐transcriptionally. In addition, we show that circular RNAs (circRNAs) NRIP1 and PTK2 interact with two RBPs, RBPMS and MLLT3, respectively. NRIP1 (Nuclear receptor‐interacting protein 1) is the host gene of the circular RNA circNRIP1, and itself is a circRNA that modulates both transcriptional activation and repression, and plays an important role in the regulation of different cancer pathways, whereas PTK2 (Protein Tyrosine Kinase 2) is the host gene of the circRNA circPTK2, and itself is also a circRNA according to.^46^, ^47^ The finding of the RBP‐circRNA pairs including RBPMS‐NRIP1, and MLLT3‐PTK2 suggests that they may also work together to modulate the expressions of a large number of genes.

### 3.4 Two RBPs interact with 17 lncRNAs on the regulatory network of pediatric AML

**FIGURE 4:**
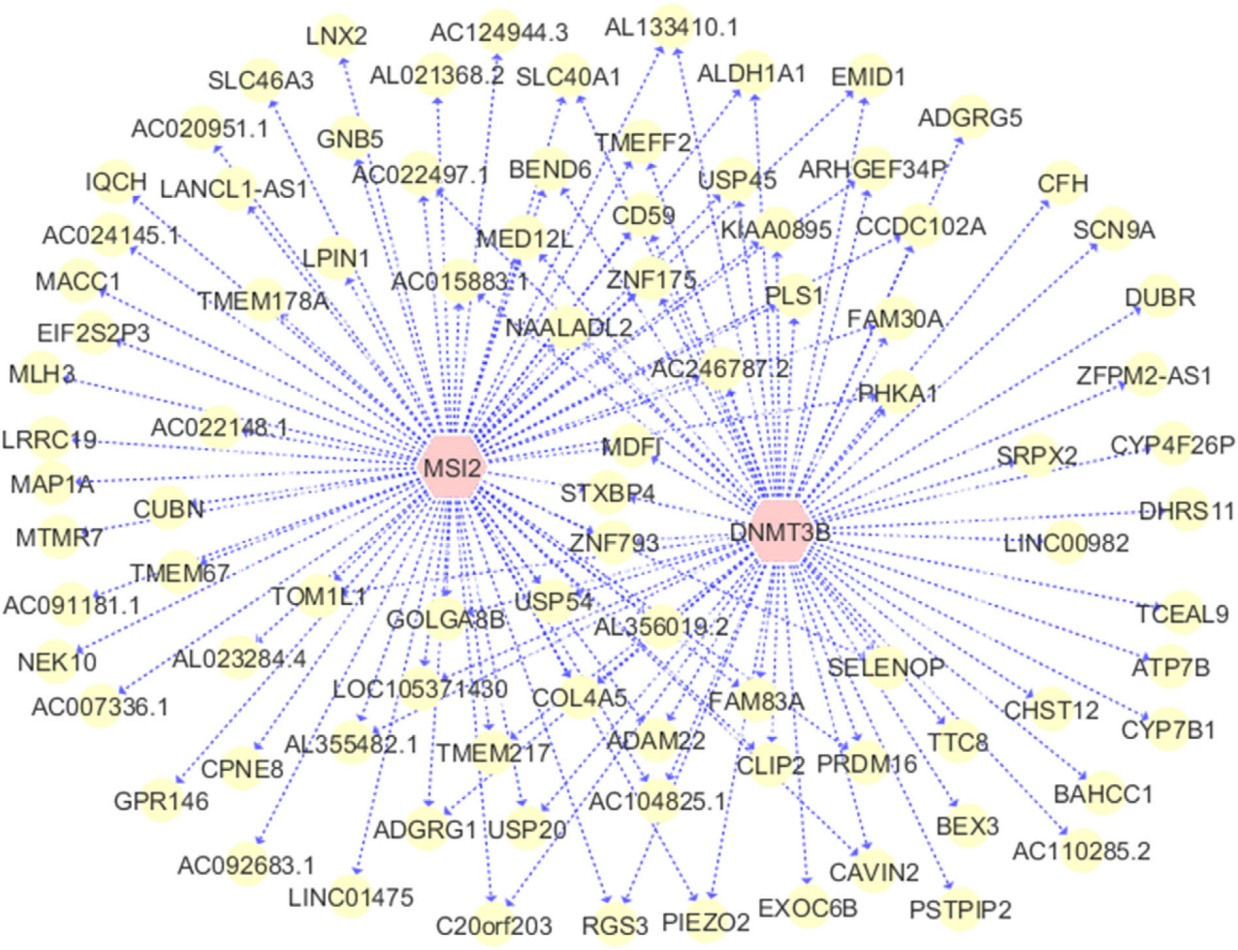
Gene regulatory network analysis with 440 upregulated genes in pediatric AML. The two pink nodes at the center are the RBPs, whereas the yellow nodes are the targeted coding and noncoding RNAs. Only RBPs regulating a large number of RNA targets are reported on the network. AML, acute myeloid leukemia; RBPs, RNA binding proteins

We construct the gene regulatory network with 440 upregulated genes in high‐risk pediatric AML with the distance correlation test, similar to what we did with adult AML previously. Again, the raw *p* value for detecting the interactions of RBPs and their targeted RNAs is set to by the Bonferroni adjustments. Two RBPs and 90 RNAs targets are identified to be associated as demonstrated in Figure 4.

MSI2 and DNMT3B are the two RBPs regulating 71 and 61 RNA targets, respectively. There are 41 commonly targeted genes regulated by both MSI2 and DNMT3B, suggesting that MSI2 functions together with DNMT3B to modulate 41 upregulated RNA targets in pediatric AML. Interestingly, MSI2 and DNMT3B are also interact with 17 long noncoding RNAs (lncRNAs) including AC007336.1, AC024145.1, AC092683.1, AC104825.1, AC246787.2, AL021368.2, AL023284.4, AL133410.1, AL355482.1, AL356019.2, CYP4F26P, DUBR, FAM30A, LANCL1‐AS1, LINC00982, LINC01475, and ZFPM2‐AS1. Out of 17 lncRNAs, 5 of them are regulated by both MSI2 and DNMT3B, including AC104825.1, AC246787.2, AL133410.1, AL355482.1, and FAM30A. lncRNAs are important regulators of gene expression, and are involved in various biological functions. We demonstrate that the 17 lncRNAs are upregulated in high‐risk pediatric AML, and function together with MSI2 and DNMT3B to regulate a large number of genes. Although both RBPs and ncRNAs are known to be the major regulators of transcriptional processes, their functions in AML have been investigated separately.^17^, ^20^ To the best of our knowledge, the functional mechanisms and causal directions between RBPs and ncRNAs in AMLs have not been explored before. The RBP‐ncRNA pairs we discovered may provide potential therapeutic targets for AML.

### 3.5 The prognostic significance of RBPMS‐NRIP1 and MLLT3‐PTK2 pairs in adult AML

**FIGURE 5:**
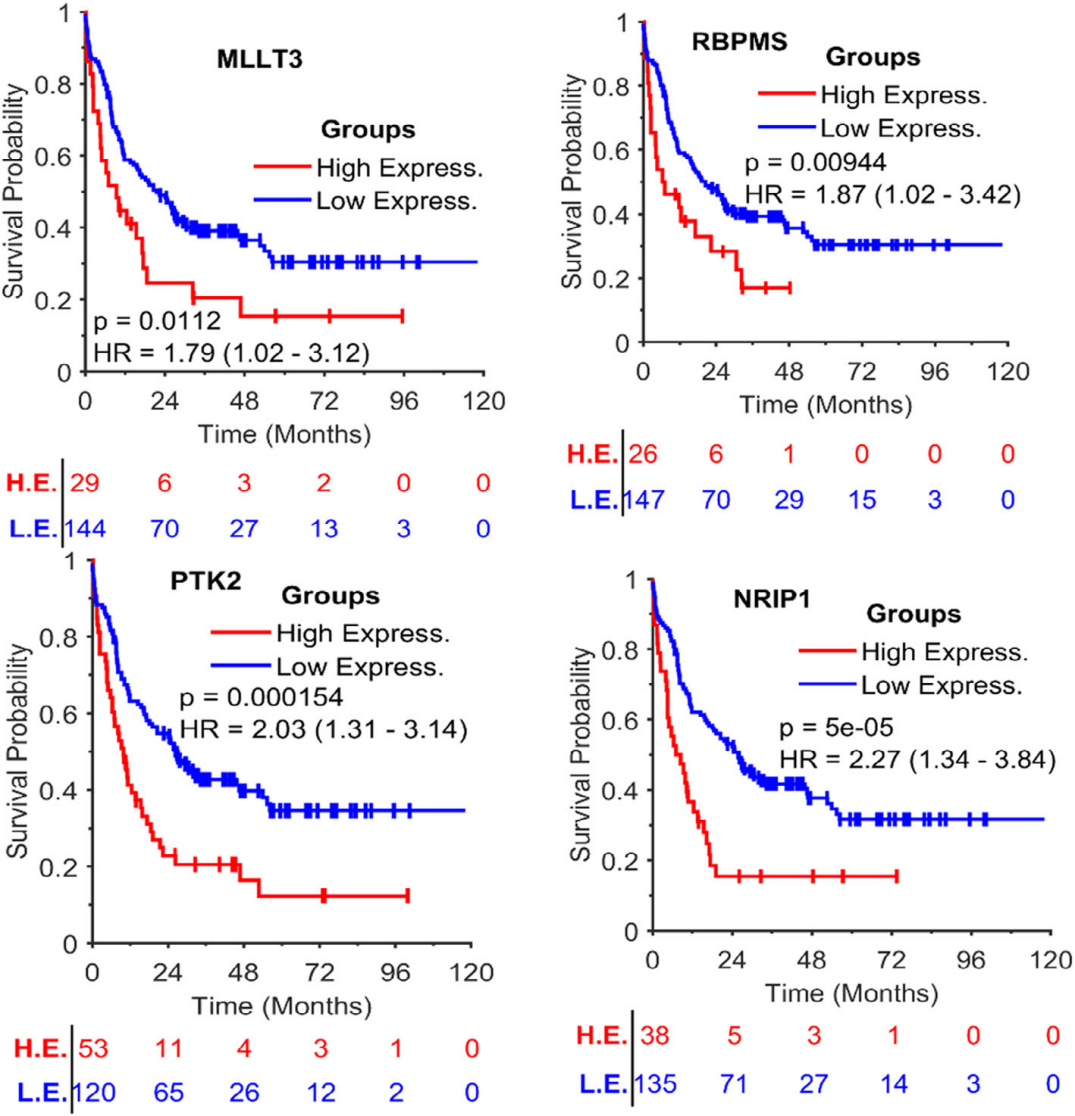
Kaplan Meier curves for the overall survival of the two RNA binding proteins and their circRNA targets. Upper Left: Overall survival for MLLT1; Upper Right: Overall survival for RBPMS; Bottom Left: Overall survival for PTK2; and Bottom Right: Overall survival for NRIP1. Where H.E. denotes the subgroup of high expression, and L.E. denotes the subgroup with low expression. The numbers below each subplot are the number of subjects at risk

Both RBPs and ncRNAs play pivotal roles in the process of gene expression, RNA maturation, and protein synthesis, and control gene expression and disease progression.^12^, ^30^ The identified RBP‐ncRNA interaction pairs may indicate that they control all the post‐transcriptional events in the cell together. Therefore, we estimate the prognostic significance of three RBPs, three snoRNAs, and two circRNAs in adult AML with log‐rank test, and identify two RBPs, MLLT3 and RBPMS, and their circRNA targets are associated with overall survival (OS). The Kaplan Meier curves are in Figure 5.

Figure 5 shows that MLLT3, RBPMS, PTK2, and NRIP1 are prognostic significance (*p* < .02), and the high expression of the four genes are associated with overall survival of the patients.

### 3.6 Two RBPs and 13 lncRNAs are associated with the overall survival of pediatric AML

We estimate the overall survival for RBPs and their lncRNA targets with univariate Cox regression. The statistically significant RBPs and targeted lncRNAs are reported in Table 1.

**TABLE 1:** Statistically significant RBPs and lncRNAs for overall survival with Cox regression

Gene ID	Beta value	Hazard ratio	Confidence interval	*p*‐Values
DNMT3B	0.261	1.298	(1.161, 1.451)	4.91E‐06
MSI2	0.282	1.325	(1.134, 1.549)	0.0004
CYP4F26P	0.133	1.143	(1.078, 1.212)	8.17E‐06
AL021368.2	0.379	1.461	(1.192, 1.790)	0.00026
AC104825.1	0.276	1.318	(1.133, 1.532)	0.00034
AC092683.1	0.254	1.289	(1.116, 1.489)	0.00056
AL133410.1	0.219	1.245	(1.086, 1.428)	0.00165
DUBR	0.143	1.153	(1.053, 1.263)	0.00219
LINC01475	0.078	1.081	(1.025, 1.141)	0.00448
AL355482.1	0.109	1.116	(1.034, 1.203)	0.00452
LANCL1‐AS1	0.173	1.189	(1.053, 1.342)	0.00506
AC007336.1	0.181	1.199	(1.050, 1.369)	0.00753
LINC00982	0.071	1.073	(1.017, 1.133)	0.01011
AL356019.2	0.197	1.218	(1.047, 1.416)	0.01049
AL023284.4	0.091	1.096	(1.011, 1.187)	0.02567

*Note*: The gene ID, estimated coefficients (Beta), hazard ratio, and its confidence interval, and *p*‐values are reported from the left to right on the table. The top two genes are RBPs, whereas the remaining genes are lncRNAs.

Abbreviation: RBPs, RNA binding proteins.

Table 1 demonstrates that the two RBPs, and 13 out of 17 lncRNAs are associated with patient overall *p*‐values (*p* ≤ 0.05) with Cox regression. All the coefficients are positive suggesting that subjects with higher expression have the poorer overall survival. MSI2 (RNA‐binding protein Musashi homolog 2) is known to be an important regulator in cancer initiation, progression, and drug resistance,^48^ whereas DNMT3B (DNA Methyltransferase 3 Beta) mediates DNA methylation, and play a crucial role in in hematopoietic stem cells.^43^ We not only discover that high expression MSI2 and DNMT3B are associated with inferior overall survival, but also find that the majority of lncRNAs correlated with MSI2 and DNMT3B are also prognostically important in pediatric AML. The lncRNAs may act as cofactors of MSI2 and DNMT3B, and have an important regulatory role in various molecular processes.

## 4 DISCUSSIONS

Pediatric and adult AML is known to be genetically different diseases.^2^, ^3^ We confirm the finding through transcriptomic analysis of the RNA‐seq data in TCGA and TARGET cohorts. By comparing the upregulated genes in high‐risk pediatric and adult AML, we discover that there are less than 5% commonly upregulated genes in both cohorts. We further demonstrate the molecular and functional differences with gene enrichment analysis. The enriched pathways and molecular functions of the upregulated genes are also distinct in childhood and adult AMLs.

RBPs regulate RNA processing at different levels including localization, translation, and stability. They are the key regulators in post‐transcriptional control of gene expression. We identify 18 and 8 RBPs that are upregulated in high‐risk adult and childhood AMLs, respectively. Only two RBPs including DNMT3B and NYNRIN are in both TCGA and TARGET cohorts, further suggesting the genetic differences between adult and pediatric AMLs. The abnormally expressed RPBs in high‐risk AML may have clinical implications and may serve as potential therapeutic targets for HR‐NB.

RBPs regulate coding and noncoding RNAs, and involve combinatorial and dynamic interactions with other genes. Constructing a RBP‐based gene regulatory network will provide novel insight into their molecular functions. Particularly, RBPs with a large number of RNA targets act as a master regulator for high‐risk AML, and may have a great influence over the expression differences in the target genes. We propose a novel statistical correlation test for gene regulatory network construction, and identify RBPs associated with a large number of upregulated genes in high‐risk adult and pediatric AML. The proposed method measures both linear and nonlinear correlations between the RBPs and other upregulated RNAs.

Out of 18 upregulated RBPs in high‐risk adult AML, we discover three RBPs including MLLT3, RBPMS and PTRF with 103 upregulated RNA targets. MLLT3 (MLLT3 Super Elongation Complex Subunit) is also known as AF9, and acts as a crucial regulator of human hematopoietic stem cells (HSCs). It plays a key role in HSC maintenance by preserving, rather than conferring, HSC stemnes.^49^ On the other hand, RBPMS (RNA‐binding protein with multiple splicing) acts as a coactivator of transcriptional activity, whereas PTRF (Polymerase I and transcript release factor) is also known as Caveolae Associated Protein 1 (CAVIN1), and plays a key role in the formation of the ribosomal transcriptional loop. The role of RBPMS and PTRF is less studied than MLLT3 in adult AML. The three RBPs are upregulated in high‐risk adult AML, and control 103 RNA targets together.

In contrast, two out of eight different RBPs including MSI2 and DNMT3B are discovered to be upregulated and together with 90 RNA targets in pediatric AML. Both MSI2 (RNA‐binding protein Musashi‐2) and DNMT3B (DNA Methyltransferase 3 Beta) have been investigated in AML independently,^43^, ^48^, ^50^ and higher expression of both RBPs are associated with poorer survival. However, the interactions of MSI2 and DNMT3B are not well studied. Through regulatory network analysis, we further show that MSI2 and DNMT3B may function together and co‐modulate 41 upregulated RNA targets in high‐risk pediatric AML.

Both RBPs and ncRNAs are known to be the major regulators of transcriptional processes and mediate RNA expression post‐transcriptionally. Dysregulation of ncRNA and RBPs contributes to cancer progression.^17^, ^20^ However, the interactions between RBPs and ncRNAs for adult and pediatric AMLs have not been explored. Distinct RBP and ncRNA pairs are identified to be associated for adult and pediatric AMLs, respectively. With the TCGA cohort, three snoRNAs (SNORD116‐4, SNORD116‐20, and SNORD116‐28) and one circRNA (PTK2) are correlated with MLLT3, and another circRNA, NRIP1, is associated with RBPMS in adult AML, whereas t7 lncRNAs are discovered to be regulated by two RBPs, MSI2 and DNMT3B, together in pediatric AML, suggesting distinct driver genes in adult and childhood AMLs. Furthermore, we discover that two RBPs, MLLT3 and RBPMS, and their circRNA targets PTK2 and NRIP are significantly associated with the OS of adult AML, whereas two different RBPs, MSI2 and DNMT3B, and 13 (out of 17) of lncRNA targets are prognostically significant in childhood AML, providing potential therapeutic targets for AML.

## 5 CONCLUSIONS

Although it is well known that pediatric and adult AMLs are genetically distinct diseases, the driver genes for high‐risk pediatric and adult AMLs are still not fully understood. Particularly, not much is known yet about the interactions between RBPs and noncoding RNA in either adult or pediatric AMLs. Based on the transcriptome profile of the TCGA and TARGET cohorts, we construct RBP‐based gene regulatory networks with upregulated genes and distance correlation tests. The upregulated genes and RBPs in pediatric and adult AMLs are different with little overlap. We identify 3 RBPs interacting with 3 snoRNA and 2 circRNAs bind and regulate over 100 upregulated RNA targets in high‐risk adult AML, whereas the other two RBPs functioning with 17 lncRNAs together control over 90 upregulated RNA targets in pediatric AML. Prognostically significant RBPs, circRNAs, and lncRNAs are identified for adult and pediatric AMLs, respectively. Exploring the regulations and interactions between RBPs and ncRNAs allows better understanding of molecular mechanisms underlying adult and pediatric AMLs, and helps to identify novel RBPs and ncRNAs therapeutic targets.

## CONFLICT OF INTEREST

The authors declare no conflict of interest.

## AUTHOR CONTRIBUTIONS

*Designed the Study, Performed the Data Analysis, and Wrote the Manuscript*, Z.L.; *Participated in the Project, Made Suggestions for Improvement, and Revised the Manuscript Critically*, V.S.S. and H.‐G.W. All authors have read and agreed to the published version of the manuscript.

## ETHICS STATEMENT

This is a secondary analysis of public available data. No human subjects are involved, and no ethical approval is required.

## Supporting information


**TABLE S1** Supplementary File

## Data Availability

The datasets analyzed for this study can be found in the public web sites including TCGA (https://www.cancer.gov/about‐nci/organization/ccg/research/structural‐genomics/tcga) and TARGET (https://www.cancer.gov/about‐nci/organization/ccg/research/structural‐genomics/tcga).
